# Theoretical study of cellulose II nanocrystals with different exposed facets

**DOI:** 10.1038/s41598-021-01438-5

**Published:** 2021-11-08

**Authors:** Can Leng, Kenli Li, Zean Tian, Yubing Si, Huang Huang, Junfeng Li, Jie Liu, Wei-Qing Huang, Keqin Li

**Affiliations:** 1 Science and Technology on Parallel and Distributed Processing Laboratory, National University of Defense Technology, Changsha, 410073 China; 2 Laboratory of Software Engineering for Complex Systems, National University of Defense Technology, Changsha, 410073 China; 3 National Supercomputer Center in Changsha, Changsha, 410082 China; 4 College of Computer Science and Electronic Engineering, Hunan University, Changsha, 410082 China; 5 College of Chemistry, Zhengzhou University, Zhengzhou, 450001 China; 6 College of Chemistry and Chemical Engineering, and Henan Key Laboratory of Function-Oriented Porous Materials, Luoyang Normal University, Luoyang, 471934 China; 7 Department of Applied Physics, School of Physics and Electronics, Hunan University, Changsha, 410082 China; 8 Department of Computer Science, State University of New York, New Paltz, NY 12561 USA

**Keywords:** Computational chemistry, Electronic properties and materials, Nanoparticles, Molecular dynamics, Structure prediction

## Abstract

Derived from the most abundant natural polymer, cellulose nanocrystal materials have attracted attention in recent decades due to their chemical and mechanical properties. However, still unclear is the influence of different exposed facets of the cellulose nanocrystals on the physicochemical properties. Herein, we first designed cellulose II nanocrystals with different exposed facets, the hydroxymethyl conformations distribution, hydrogen bond (HB) analysis, as well as the relative structural stability of these models (including crystal facets {A, B, O} and Type-A models vary in size) are theoretically investigated. The results reveal that the HB network of terminal anhydroglucose depends on the adjacent chain’s contact sites in nanocrystals exposed with different facets. Compared to nanocrystals exposed with inclined facet, these exposed with flat facet tend to be the most stable. Therefore, the strategy of tuning exposed crystal facets will guide the design of novel cellulose nanocrystals with various physicochemical properties.

## Introduction

As an environmentally friendly and low-cost material, cellulose has been widely used in medicinal, environmental, and separation technologies in recent decades^1–3^. Cellulose is composed by linear chains of ringed glucose in which the highly ordered and disordered structures can form crystalline and amorphous-like, respectively. In general, there are four distinct crystalline celluloses in two groups of the natural fiber on earth (cellulose I), and the man-made fibers from synthesis (cellulose II, III, and IV)^4^. Cellulose II that the chains polarity is antiparallel to each other^5–7^ is derived from celluloseI^8^ by either mercerization or regeneration processes. Among the chains opposite to each other in cellulose II, the outer one is usually assigned as Origin (O) and the inner one as Center (C), respectively^9^. Moreover, the hydroxymethyl group structure of cellulose II is different from cellulose I. Contrary to the trans-gauche (tg) conformation of celluloseI^10,11^, cellulose II adopts a gauche−trans (gt) conformation^5–7^, which can be diversified in gauche–gauche (gg) conformation^12^ in some small crystal morphologies.

Cellulose nanocrystals refer to the size of cellulose crystalline on the nanoscale. The relative stability of cellulose nanocrystals varies with their crystalline forms. For example, Navarrete‑López1 et al.^13^ reported that cellulose I*β* is more stable than cellulose I*α* due to the weak interaction between layer chain arrangement. Goldberg et al.^14^ have studied the quantitative order of stability for cellulose through experimental methods and showed that the stability of cellulose III is higher than cellulose II, followed by cellulose I*β*. On the other hand, the cellulose nanocrystals with different shapes and sizes have different relative stability due to the energy discrepancy of different facets and inter-sheet interactions (such as hydrophobic interactions and weak hydrogen bonds). For instance, it has been demonstrated that the 234,432 chain arrangement based on 6 layers cellulose nanocrystals is more stable than the 34,443 chain arrangement based on 5-layer with the same 18 cellulose polymer chains^15^. In order to explore the mechanism of the relative stability of cellulose nanocrystals, a series of theoretical study has been conducted^16–18^ in recent years. For instance, Yui studied^19^ the structural stability of the solvated cellulose III_I_ crystal through molecular dynamics (MD) simulation and confirmed the metastable properties of cellulose III_I_ by the reversible transition to the cellulose I*β* due to the quarter staggered structure along the axis direction. The structural stability of the finite molecular chain cellulose I*α*, I*β*, II, and IIII by density functional theory (DFT) by Uto et al.^20^, indicated that the stacking and crystal transformation of cellulose crystals were mainly affected by the different stacking methods of the cellulose backbone. A calculation^21^ (mixed MD with DFT) was adopted to investigate the relative structural energy of native plant cellulose with different shapes to evaluate its relative stability. However, due to the computational bottleneck caused by the Kohn–Sham (KS) equation^22,23^, conventional DFT calculations are usually limited to a few hundred atoms. Thanks to the development of semi-empirical methods in recent years^24–26^, it is possible to conduct simulations for a large molecular system including thousands of atoms.

Hydrogen bond (HB) is an essential factor that influences the physicochemical properties of cellulose nanocrystals^27–29^. In the crystalline cellulose, three-dimensional hydrogen-bonded networks formed by hydroxyl groups can influence the cellulose backbone comprised by large number of covalent bonds (i.e., C–O, C–H, O–H). Particularly, these HB linkages play an important role in chain stiffness and stability of the cellulose^30,31^. Experiments have shown that HB networks can be regulated and resulted in different crystal morphologies^10,32,33^. For example, different HB networks can be formed on some anhydroglucose residues not fully confirmed, which are speculated by Idström et al.^34^, to be related to different exposed facets of regenerated cellulose. Therefore, different crystal morphologies can be obtained by regulating the HB network, thus, resulting in different properties. Unfortunately, these HBs structure of cellulose is difficult to be directly determined experimentally because of the inaccuracy of hydrogen atoms using classical techniques such as X-rays and neutron diffraction. Even worse, due to some cellulose's small diameter with partially disordered regions on the surface, it is currently difficult to accurately study the HB distance^35^ and hydroxymethyl groups where each glucose residue unit rotates with respect to the ring atoms^36^ through experimental techniques. Therefore, it is challenging to design novel cellulose nanocrystals exposed different facets, which are relatively stable by modulating HBs to obtain desirable specific properties and functions.

## Results and discussion

In this work, we first theoretically designed several cellulose II nanocrystals that expose (0 0 1), $(\overline{{1}} \; {1}\; {2)}$, and $(\overline{{2}} \;{2}\;{1)}$ facets respectively, by tuning the size of chains and/or HBs. The MD simulation and DFT calculations are carried out to investigate the hydroxymethyl structures, HBs properties and their relative structural stability. We find that different contact sites between the ends of the adjacent chains can affect the HB formation of terminal anhydroglucose. The relative stability of cellulose II nanocrystals is dependent on their inclined facets. The results obtained in this work are useful to synthesize novel celluloses with different physicochemical properties.

**Figure 1:**
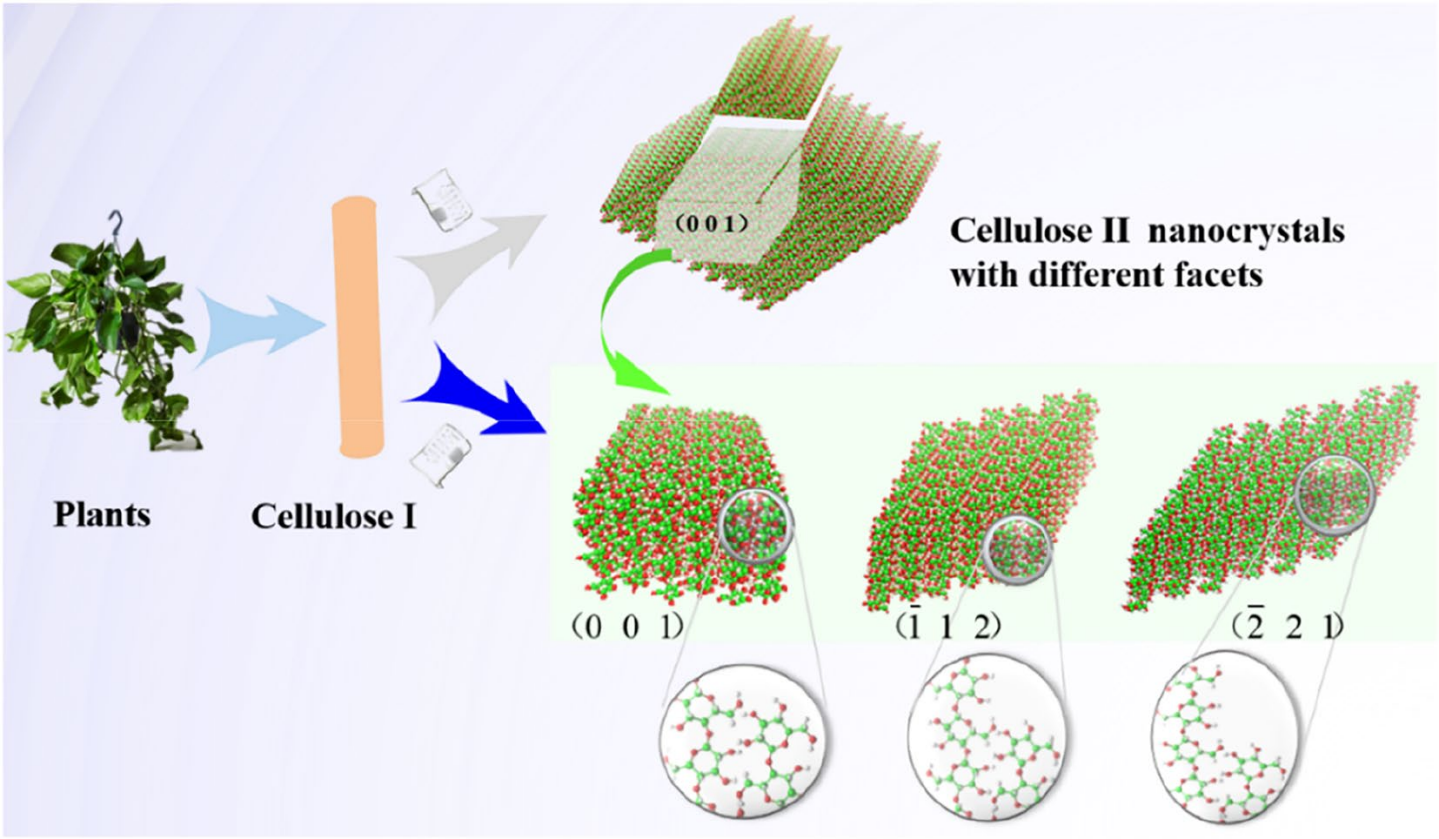
Cellulose II nanocrystals with different exposed facets. Cellulose I represent the native material in plants, from which Cellulose II can be synthesized. The facet (0 0 1) is the primary crystal facets of cellulose II nanocrystals, $(\overline{{1}} \;{1}\;{2)}$ and $(\overline{{2}} \;{2}\;{1)}$ are the inclined facets of cellulose II.

**Figure 2:**
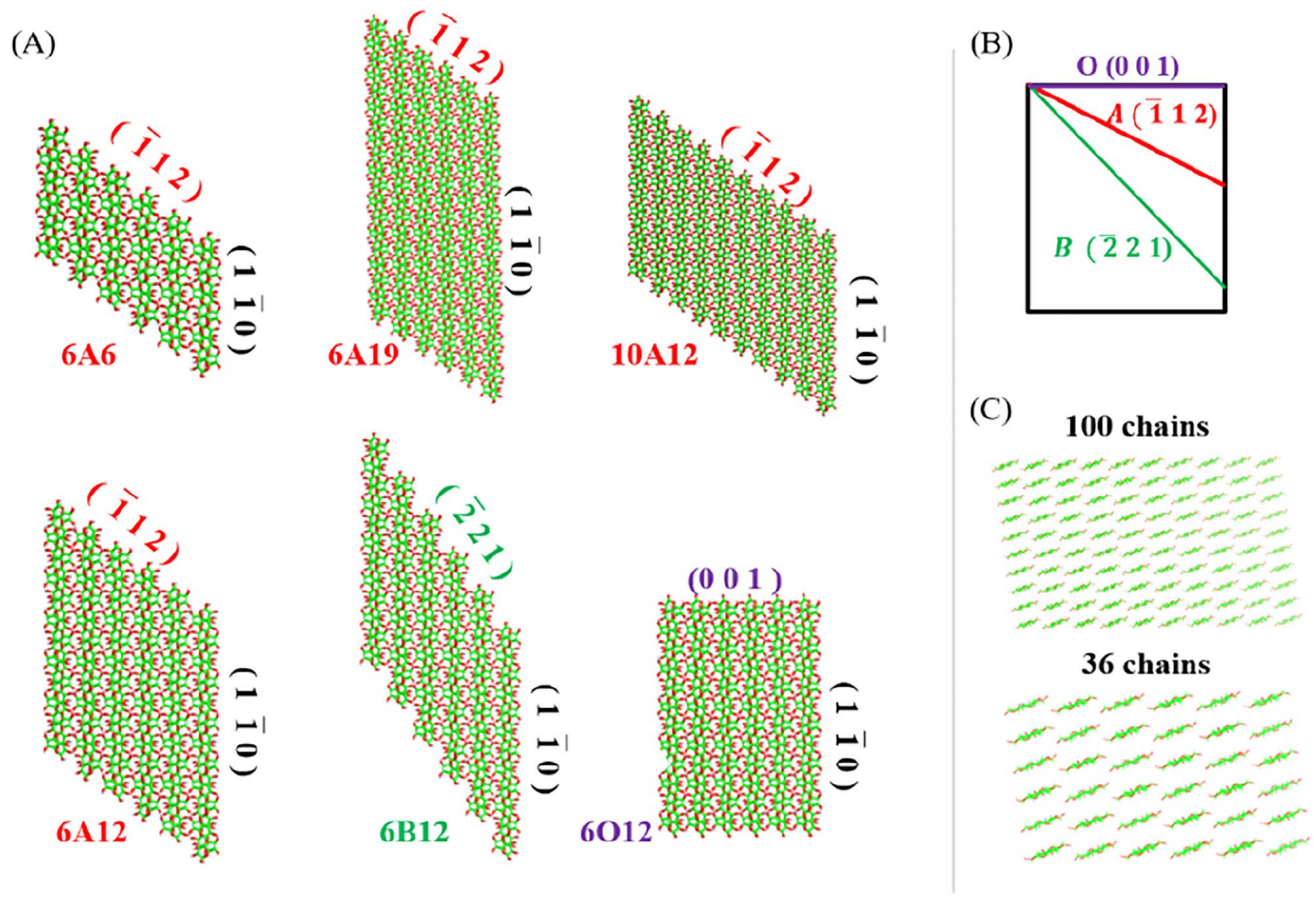
The structure and crystal facets of cellulose II nanocrystals. (**A**) The geometry of six types of cellulose II nanocrystal models. 6A6, 6A12, 6A19, and 10A12 denote 6 × 6 × 6, 6 × 6 × 12, 6 × 6 × 19, and 10 × 10 × 12 the crystal models with the crystal facets $(\overline{{1}} \; {1}\; {2)}$, respectively. 6B12 and 6O12 are 6 × 6 × 12 crystal models with $(\overline{{2}} \; {2}\; {1)}$ and (0 0 1) as crystal facets respectively; (**B**) Three types of inclined crystal facets. (C) 6 × 6 and 10 × 10 are the chain numbers of the ab base plane projections.

The six cellulose II nanocrystal models are constructed based on a toolkit developed by Gomes^37^. The model parameters are shown in Table 1, and Fig. 1 shows the detailed structures. In these nanocrystal models, there are three types of the exposed facets with the index of $(\overline{{1}} {1} {2)}$, $(\overline{{2}} {2} {1)}$, and (0 0 1), labeled as A, B, and O in the paper for convenience. The facets A and B are derived from O that is the usual crystal facet (0 0 1), as shown in Fig. 2A,B. Then a nanocrystal including *i* × *i* chains (see Fig. 2C) can be represented as “*i*L*j*”, where *j* is the Degree of Polymerization (DP) of the chains, and the letter L can be A, B, and O, as demonstrated in Fig. 2A. For convenience, a nanocrystal including A (B, or O) facet is called Type-A (B, or O) crystal, for example, if the ends of 6 × 6 chains comprise a $(\overline{{1}} \;{1}\; {2)}$ facet, and DP is 12 for each chain, it is assigned as 6A12, and it is a Type-A nanocrystal.

**Table 1:** Dimensions and number of molecules for the six types of cellulose II nanocrystal models.

The label of crystals model	Base plane dimensions	No. of chains	DP	No. of water
6A6	6 $({1}\; \overline{{1}} \; {0)}$ × 6(1 1 0) × 6 $(\overline{{1}} \; {1}\; {2})$	36	6	12,532
6A12	6 ${(1}\; \overline{{1}} \; {0)}$ × 6(1 1 0) × 12 $(\overline{{1}} \; {1}\; {2)}$	36	12	16,299
6A19	6 $({1}\; \overline{{1}} \; {0)}$ × 6(1 1 0) × 19 $(\overline{{1}} \;{1}\; {2)}$	36	19	37,444
10A12	10 ${(1}\; \overline{{1}} \;{0)}$ × 10(1 1 0) × 12 $(\overline{{1}} \; {1}\; {2)}$	100	12	45,148
6B12	6 ${(1}\; \overline{{1}} \; {0)}$ × 6(1 1 0) × 12 $(\overline{{2}} \;{2}\; {1)}$	36	12	20,576
6O12	6 ${(1}\; \overline{{1}} \;{0)}$ × 6(1 1 0) × 12 ${(0}\;{ 0}\;{ 1)}$	36	12	12,171

### Hydroxymethyl conformations analysis

**Figure 3:**
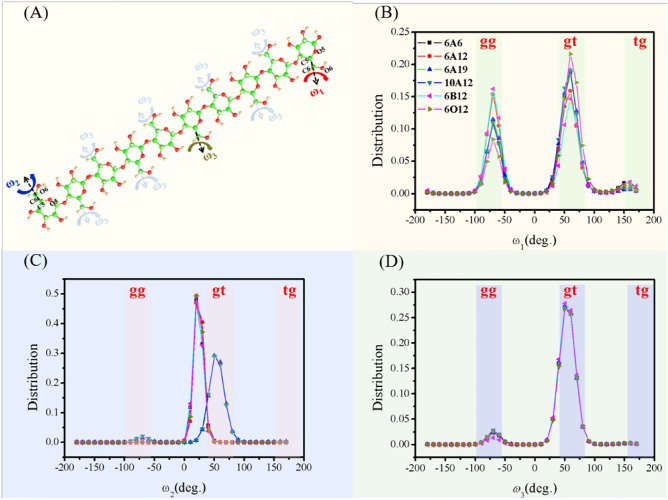
The characteristic dihedral angle O_5_-C_5_-C_6_-O_6_ distribution in the MD process. (**A**) Definition of the torsion angle parameters of the hydroxymethyl group; (**B**–**D**) are the dihedral angles *ω*_1_, *ω*_2_ and *ω*_3_ distributions of the residues extracted from the MD trajectories in the last 1 ns, respectively.

First, we focus on the influence of the cellulose II nanocrystal model on the hydroxymethyl group conformations. It is generally accepted that cellulose II has gt and gg conformations^5–7^, while the tg conformations are very rare^10^. The selected dihedral angle of the configuration is shown in Fig. 3A. There are three cases for the dihedral angle O_5_–C_5_–C_6_–O_6_ on the anhydroglucose residue. One is that O_5_ and O_6_ are facing out of the chain, represented by *ω*_1_; the second is that O5 and O6 are facing inward of the chain, represented by *ω*_2_; all others are regarded as *ω*_3_.

Figure 3B shows the distribution of the hydroxymethyl group conformations (*ω*_1_) detected in the terminal residues. Obviously, two peaks appear near − 60° (*ω*_1_ ≈ gg) and 60° (*ω*_1_ ≈ gt), which means the hydroxymethyl groups frequently rotated between gt and gg position in 6A12, 6B12, and 6O12. In general, the gt distributions of 6O12, 6A12, and 6B12 are 21.60%, 15.89%, and 14.66%, respectively, indicating the more inclined the facet, the less gt conformation for hydroxymethyl group. Opposite results can be found in the gg distribution, where 6O12, 6A12, and 6B12 are 8.39%, 16.23%, and 15.40%, respectively, indicating that the gg distribution of the hydroxymethyl group decreases significantly with the incline of the nanocrystal facet. Therefore, compared to the flat crystal facet of 6O12, an inclined crystal facet can lead to fewer gt distribution and more gg distribution of hydroxymethyl group. Furthermore, the gt distribution of 6A6, 6A19, and 10A12 are 18.74%, 19.08%, and 19.08%, respectively, slightly larger than 6A12 (15.89%). The gg distribution of 6A6, 6A19, and 10A12 are 11.16%, 11.51%, and 11.00%, respectively, which are somewhat smaller than 6A12. The current MD simulation still reproduces the minor conformations of the hydroxymethyl groups very well. For example, a minor peak also appears near tg (*ω*_1_≈180°).

Figure 3C shows the distribution of hydroxymethyl group conformations (*ω*_2_) detected in the terminal residues of the crystal model. Compared with Fig. 3B, the hydroxymethyl group of *ω*_2_ hardly rotates in gg position. What's more, there is almost no difference of hydroxymethyl conformation in 6A12, 6B12, and 6O12. Moreover, the distribution of hydroxymethyl groups is almost around 20° instead of 60° at the gt position. For Type-A models, as the chain and DP increase, the hydroxymethyl group of *ω*_2_ gradually changes from about 20° to 60°, reaching the angular distribution range of the gt conformation. The distribution of hydroxymethyl group conformations (*ω*_3_) detected in the internal anhydroglucose of the nanocrystal models is shown in Fig. 3D, where the hydroxymethyl group of *ω*_3_ is almost at gt position with less at gg position. Compared with the conformations of *ω*_2_ and *ω*_3_ only at the gt position, the hydroxymethyl conformations of *ω*_1_ are mainly distributed at the gg and gt positions, which can be inferred the possibility of different chemical properties at the chain end positions.

### Hydrogen bond analysis

The HB network can be regulated to obtain different nanocrystal facets; therefore, different properties of the reducing chain end can be obtained on different exposed facets, which may be necessary for subsequent chemical modification and specific potential applications. Hence, we are motivated to look for HBs differences in these nanocrystal models. Besides the usual HBs of cellulose, i.e., the O_2_H⋯O_6_ and O_6_H⋯O_6_^33,38^, more types of HBs were investigated. To obtain more detailed information about HBs in these models, the HBs strength is further calculated by optimizing the geometries in the highest HBs fraction. As represented in Supplementary Table S1, it manifests the average HBs analysis of the terminal residues in the six nanocrystal models. The average fraction $\overline{F}$, O–O distance and angle are averaged according to the HBs fraction (only the fraction large than 60% are considered). The fraction F of the *j*th HB type in the *i*th sample (there are eight HB types and 5000 sample in total) is defined in the following equation (Eq):

1 $$F_{j}^{i} = \frac{{x_{j}^{i} }}{{\sum\limits_{j} {x_{j}^{i} } }}$$

The highest HBs fraction *F*_max_ of the *j*th HB type in the *i*th sample is defined as:

2 $$F\left( {j,max} \right) = max\left\{ {F_{j}^{i} , \, i = 1,5000} \right\}$$

The average fraction $\overline{F}$ of the *j*th HB type in all samples is defined as:

3 $$\overline{F}_{j} = \frac{{\sum\limits_{i = 1}^{m} {(F_{j}^{i} \cdot N_{i} )} }}{{\sum\limits_{i = 1}^{m} {N_{i} } }}$$

Since *N*_i_ is basically the same, let $N_{i} \approx n$, then:

4 $$\overline{F}_{j} = \frac{{\sum\limits_{i = 1}^{m} {(F_{j}^{i} \cdot n)} }}{{\sum\limits_{i = 1}^{m} n }} = \frac{{\sum\limits_{i = 1}^{m} {(F_{j}^{i} \cdot n)} }}{m \cdot n} = \frac{{\sum\limits_{i = 1}^{m} {F_{j}^{i} } }}{m}$$

Hence $\overline{F}$ in Supplementary Table S1 is derived from Eqs. (1), (2), (3), and (4). In Supplementary Table S1, the HBs are O_6_H⋯O_2_, O_2_H⋯O_2_, O_6_H⋯O_6_, O_3_H⋯O_6_, O_2_H⋯O_6_, O_6_H⋯O_3_, O_2_H⋯O_3,_ and O_3_H⋯O_5_. It can be seen that O_6_ and O_2_ are most favorable in the HBs formation. We first consider the effects of different nanocrystal models 6A12, 6O12, and 6B12 on HBs. Supplementary Fig. S1A shows that 6O12 forms slightly more HBs than 6A12 and 6B12. Meanwhile, the $\overline{F}$ of different HBs types in Supplementary Fig. S1B indicates that the HB fraction and type in 6A12 are maximum compared with 6O12 and 6B12. What’s more, it seems less favorable for 6B12 to form HBs because it has only four types of HBs compared with 6A12 and 6O12. We speculate that the HB interaction between the adjacent terminal residues will first increase and then decreases with the increase of the inclined crystal plane. Therefore, it can be inferred that HBs are more likely to be formed in 6O12 and 6A12 than in 6B12.

According to Supplementary Fig. S1C, the change of DP rarely affects the number of HBs in the Type-A nanocrystal models. More precisely, Supplementary Fig. S1D shows the types of HBs and the $\overline{F}$ in different DP models, and it revealed that O_6_H⋯O_2_ has the most frequent appearance when DP increased from 6 to 19, followed by O_2_H⋯O_2_, O_2_H⋯O_6,_ and O_6_H⋯O_6_. Alternatively, O_3_H⋯O_5_ seems to be a little impacted by the DP changes. In 6A6, O_3_H⋯O_4_ is gradually replaced by O_6_H···O_3_ as DP increases, indicating that the HBs involving O_4_ are not easy to form in Type-A nanocrystal models. Hence, the increase of DP in Type-A models will cause the change of HB type. As shown in Supplementary Fig. S1E and F, the increasing chain from 6A12 to 10A12 brings a distinct HB number increase from 40 to 120 with the $\overline{F}$ similar to each other. In addition, O_6_H···O_3_ is found in 6A12, and disappeared in 10A12.

**Figure 4:**
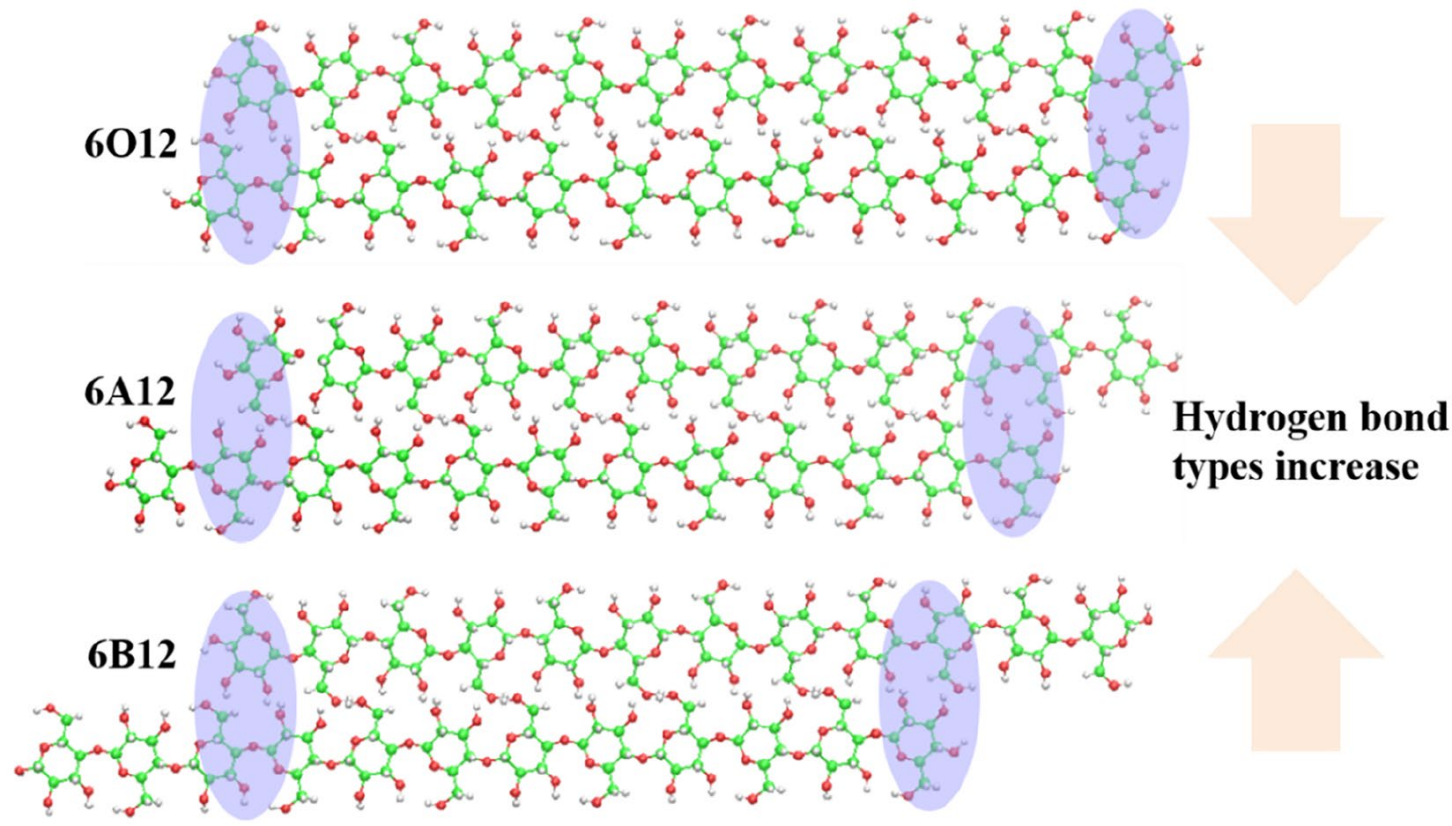
The influence of inclined facet on the hydrogen bond formation. Neighbor chain weak interaction of the terminal anhydroglucose varies in inclined crystal facets for cellulose II nanocrystal models.

Therefore, the HBs network changes with the interaction of terminal anhydroglucose between two adjacent chains on the exposed facet. In other words, the HB network can be formed by the adjacent anhydroglucose at the terminal chain changes with the inclined of the crystal facet. Hence, by regulating these HBs networks formed at the end of chains, different nanocrystal facets characteristics can be obtained. As shown in Fig. 4, to obtain different types of HBs, the exposed facet composed of chain ends should be designed with an appropriate slope, which can be reflected in the terminal anhydroglucose position. For the convenience of description, we define terminal, proximal, and distal position as the first, the second and remaining anhydroglucoses of a chain, respectively.

As shown in Fig. 4, the blue area represents the contact position between the neighbor anhydroglucose. It reveals that the types of HB for 6A12 and 6O12 is greater than that of 6B12. We speculate that it is related to the contact position of adjacent terminal anhydroglucoses. On the one hand, for the terminal and proximal position, it has the smaller anhydroglucose steric hindrance and the higher gt and/or gt distribution of the terminal hydroxymethyl group than in the distal position. Hence it is more likely to form types of HB at the terminal and/or the proximal position of 6A12 and 6O12 than at the distal position of 6B12. On the other hand, the contact area of 6A12 is slightly larger than that of 6O12, leading to more contact site of 6A12 to form more types of HB.

The probability of HBs and nanocrystal models have no definite correlation, so we first identify the sample in which a certain type of HB gets the maximal probability for every model, then calculate the bond energy based on the electron charge density obtained by Eq. (5). Eight samples (the ONIOM models) are demonstrated in Supplementary Fig. S2A, where the HB with the maximal probability are marked; and the structural unit of each HBs are presented in Supplementary Fig. S2B. Table 2 lists the detailed information of these HBs in nanocrystal models, the maximal probability is defined by *F*_max_ based on Eq. (2). From the bond energy listed in Table 2, one can find that these HBs are weak to medium strength based on Emamian classification^39^. Most of the HBs’ strengths are similar to each other. For example, the bond strength of O_2_H⋯O_6_ for 6A6 6A19 and 10A12 is about 8.0 kcal·mol^−1^, which is slightly greater than that of 6A12 and 6O12. The bond energy of O_2_H⋯O_2_ and O_6_H⋯O_6_, (corresponding to the weak to medium strength) increases from 6A6, 6A12, to 6A19, indicating the increase of nanocrystals DP can enhance the strength of the two HBs. Except for 6B12, the bond energy of O_3_H⋯O_5_ is similar but slightly more extensive than that of 6A12 and 6O12. This is because its specific crystal facet that can make the related –O_3_H– end less affected by other HB. Supplementary Table S2 shows the HBs type of these nanocrystal models, which is still highly consistent with the trend of HBs increasing from Type-O and Type-B to Type-A models.

**Table 2:** Details of HBs in the sample where the fraction of an individual HB is highest among all samples for all the six models.

Models	Type	*F*_max_	Dist/Å	Ang/°	*ρ*(r)	*E*_HB_/ kcal·mol^−1^
6A6	O_2_H⋯O_2_	0.925	2.748	162.870	0.035	− 7.036
	O_6_H⋯O_2_	0.950	2.743	160.517	0.026	− 4.969
	O_6_H⋯O_6_	0.923	2.767	162.871	0.036	− 7.218
	O_2_H⋯O_6_	0.913	2.757	161.001	0.043	− 8.901
	O_3_H⋯O_5_	0.938	2.738	160.252	0.025	− 4.907
	O_3_H⋯O_4_	0.663	2.771	160.359	0.035	− 7.161
6A12	O_2_H⋯O_2_	0.930	2.747	162.849	0.038	− 7.732
	O_6_H⋯O_2_	0.956	2.749	161.395	0.033	− 6.607
	O_6_H⋯O_6_	0.924	2.765	163.360	0.037	− 7.517
	O_2_H⋯O_6_	0.891	2.754	159.548	0.023	− 4.291
	O_3_H⋯O_5_	0.942	2.728	159.264	0.025	− 4.725
	O_6_H⋯O_3_	0.671	2.767	162.158	0.038	− 7.760
6A19	O_2_H ⋯O_2_	0.960	2.762	164.572	0.039	− 8.025
	O_6_H⋯O_2_	0.976	2.736	161.872	0.018	− 3.306
	O_6_H⋯O_6_	0.923	2.761	162.411	0.038	− 7.769
	O_2_H⋯O_6_	0.984	2.722	163.884	0.040	− 8.064
	O_3_H⋯O_5_	0.974	2.707	160.031	0.024	− 4.554
	O_6_H⋯O_3_	0.634	2.804	159.806	0.039	− 8.034
10A12	O_2_H⋯O_2_	0.926	2.752	162.679	0.031	− 6.131
	O_6_H⋯O_2_	0.962	2.746	161.647	0.037	− 7.452
	O_6_H⋯O_6_	0.924	2.767	163.299	0.034	− 6.918
	O_2_H⋯O_6_	0.914	2.752	160.441	0.040	− 8.223
	O_3_H⋯O_5_	0.948	2.731	159.488	0.027	− 5.237
6B12	O_2_H⋯O_2_	0.914	2.748	162.806	0.030	− 6.029
	O_6_H⋯O_2_	0.921	2.750	160.769	0.023	− 4.414
	O_2_H⋯O_6_	0.926	2.752	161.920	0.033	− 6.646
	O_3_H⋯O_5_	0.930	2.732	159.522	0.042	− 8.586
6O12	O_6_H⋯O_2_	0.844	2.756	161.307	0.035	− 7.126
	O_3_H⋯O_6_	0.712	2.788	159.231	0.037	− 7.412
	O_2_H⋯O_6_	0.764	2.786	158.271	0.020	− 3.703
	O_3_H⋯O_5_	0.958	2.732	161.898	0.020	− 3.751
	O_6_H ⋯O_3_	0.872	2.747	161.186	0.026	− 5.107

The HBs type, fraction (*F*_max_) and O⋯O distance (Dist), HB angle (Ang), the electron density *ρ*(r) and corresponding HBs strength (*E*_HB_) are included.

### The stability of cellulose II nanocrystals

To quantify the stability of 6A12, 6B12, and 6O12, DFT calculations are conducted on their isolated chain of cellulose II. Generally, the lower the relative energy, the stable the structure. For an isolated chain of cellulose II nanocrystals, the relative energy is related to all possible facets in a model. Due to the antiparallel chain in cellulose II, when considering (1 1 0) facet, the corresponding (2 2 0) facet is also considered, as shown in Supplementary Fig. S3, which includes two types of chain sequence: OCOCOC and COCOCO (as described above, O represents the Origin chain, C represents the Center chain); similarly, the crystal facets (0 1 0) and (0 2 0) are also considered together, including two types of chain sequence: OOOOOO and CCCCCC.

Table 3 lists the DFT calculated energies of the three B3LYP-D3BJ DFT optimized 3 × cello-tetramer chain sheet models in which Oxygen and Carbon atoms are fixed.$\Delta E_{sheet}$ and $\Delta E_{bind}$ is defined by Eqs. (6) and (7). The BSSE counterpoise correction is estimated in vacuum to correlate the $\Delta E_{sheet}$ and $\Delta E_{bind}$ values between adjacent oligomers, which is used for further correlation of these two values in water circumstances.

**Table 3:** DFT energies of the B3LYP-D3BJ optimized 3 × cello-tetramer chain sheet models (kcal·mol^−1^ per residue).

Models		DFT energies-vacuum			DFT energies-water	
		$\Delta E^{{\text{a}}}_{sheet}$	$\Delta E_{bind}$	BSSE	$\Delta E^{{\text{a}}}_{sheet}$	$\Delta E_{bind}$
6A12	(1 1 0) + (2 2 0)	0.879	− 4.681	1.228	0.454	− 2.904
	(0 1 0) + (0 2 0)	2.435	− 2.979	0.881	1.321	− 2.010
6B12	(1 1 0) + (2 2 0)	2.872	− 3.480	0.838	1.622	− 1.902
	(0 1 0) + (0 2 0)	3.161	− 2.850	0.698	1.845	− 1.653
6O12	(1 1 0) + (2 2 0)	0.000	− 5.624	1.401	0.000	− 3.372
	(0 1 0) + (0 2 0)	1.477	− 4.023	1.142	0.400	− 3.138

^a^Relative to the plane of (1 1 0) by 6A12 model.

In vacuum, the three types of chain sheet models have different $\Delta E_{bind}$ and, consequently, different $\Delta E_{sheet}$. These $\Delta E_{bind}$ values for 6O12 structures are much lower than the rest residue. For example, for 6O12 $\Delta E_{bind}$ is − 9.647 kcal·mol^−1^ (the sum of (− 5.624) + (− 4.023)), lower than the value of 6.330 kcal·mol^−1^ (the sum of (− 3.480) + (− 2.850)) for 6B12, and that of 7.660 kcal·mol^−1^ the sum of for 6A12. The crystal facets (0 1 0) and (0 2 0) in the 6B12 model has the highest $\Delta E_{sheet}$, followed by the crystal facets (1 1 0) and (2 2 0) in 6B12 model, as well as the crystal facets (0 1 0) and (0 2 0) in 6A12 model. It can be concluded that 6O12 is the most stable nanocrystal model, corresponding to the fact that Type-O with the exposed facet (0 0 1) is the most common form of cellulose II nanocrystals.

In water, these structures become more stable, as demonstrated by the decrease of $\Delta E_{sheet}$ from − 0.4 (the minimal sheet energy gap between vacuum and water circumstance: 0.879–0.454) to − 1.3 (the maximum sheet energy gap between vacuum and water circumstance: 3.161–1.845) kcal·mol^−1^, and $\Delta E_{bind}$ from − 0.9 (the minimal bind energy gap between vacuum and water circumstance: (− 2.979)–(− 2.010)) to − 2.3 (the maximum bind energy gap between vacuum and water circumstance: (− 5.624)–(− 3.372)) kcal·mol^−1^ per residue compared to the values in vacuum. On the one hand, when the exposed facet is inclined, the adjacent chains are gradually separated from each other with the smaller contact area. Generally speaking, HB can play a role in stabilizing the structure. The structural stability from Type-O to Type-A and Type-B should have the same changing trend as the aforementioned HB quantity change, that is, first rise and then fall. However, the structural stability is not only affected by hydrogen bonds but also by other weak interactions, such as electrostatic interactions, van der Waals forces, and hydrophobic interactions, which are all related to the contact area of the neighbor chains. The larger the contact area, the weaker these interactions and the more unstable the structure. The continuously inclined facets from Type-O to Type-A and Type-B make the contact area of neighbor chains smaller, thereby weakening their weak interaction and leading to a more unstable three-dimensional structure. Therefore, the minimum energy of $\Delta E_{sheet}$ and $\Delta E_{bind}$ are still dominated by 6O12 in the different exposed cellulose II nanocrystals facets.

**Figure 5:**
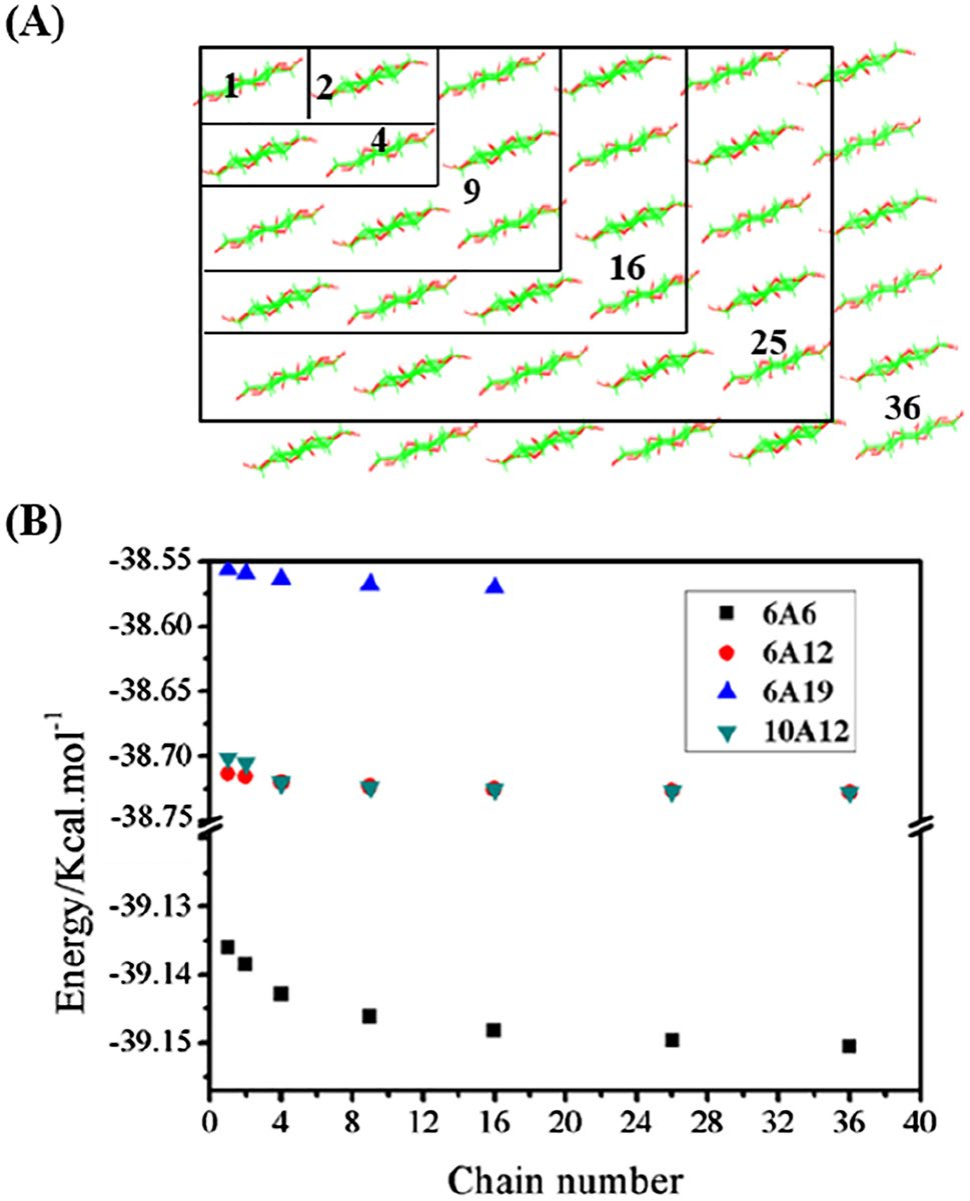
Energy changes in the extension of the sheet chain. (**A**) The crystal models of 1, 2, 4, 9, 16, 25, 36 chain numbers for 6A6, 6A12, 10A12 and 1, 2, 4, 9, 16 chain numbers for 6A19; (**B**) energy comparison of Type-A models of different sizes.

When considering the length (6A6, 6A12, 6A19) and number (6A12 and 10A12) of chains for Type-A crystal, we use the extended semi-empirical scheme GFN2-xTB. Figure 5A shows the spacious distribution of chains in different models containing 2 or *k*^2^ (*k* = 1, 2, 3, 4, 5, and 6) chains. Figure 5B reveals that 6A6 is the most stable (with the lowest energy); the bigger the DP (the length of chain), the higher the energy, and hence the lower the stability. Furthermore, the increased rate of energy decreases with the increase of DP. Therefore, the structure will eventually stabilize, and more extension of DP will no longer affect its total energy. For the effect of the number of chains on the stability, one can find that 6A12 and 10A12 have almost equal energy (stability); so that the effect of the number of chains on stability can be ignored. Therefore, DP plays a significant impact on structural stability.

## Conclusion

In summary, we have studied the properties of six cellulose II nanocrystal models via MD simulation and DFT calculations. The results revealed an obviously gt and gg position at *ω*_1_ hydroxymethyl groups and mainly gt position at *ω*_2_ and *ω*_3_ hydroxymethyl groups. The HB types and quantities are further discussed. It turns out a slight superiority of the HB type for 6A12 compared with 6O12, and 6B12 has the least HB type, which can be attributed to the different contact sites of the anhydroglucose at the ends of adjacent chains. The HB strengths investigated by using the newly developed method are in the range of weak to medium, which provides an intuitive reference guide to further research on the content of HBs in organic polymers. A study on the relative structural stability indicated that the nanocrystal with a flat exposed facet is most stable, and the stability gradually decreases when the facets become more inclined. The chain length rather than the number of sidechains is more important for structural stability in Type-A nanocrystals. These results theoretically indicate that properties can be tuned by unambiguously designing the cellulose II nanocrystal facets. These findings will guide further research on syntheses novel celluloses with different physicochemical properties.

## Methods

All 6 models (6A12, 6B12, 6O12, 6A6, 6A19, and 10A12) are put into a periodic box to ensure a complete water box environment. A partial optimization of the TIP3P water configurations is minimized, followed by a full optimization of the whole system's solvated crystal model. The structures of the nanocrystal models are fixed constrained by a force of 500 kcal·mol^−1^. Then water configurations are equilibrated using the NVT ensemble with a gradual increase in temperature from 20 to 300 K in 300 ps, followed by the constant pressure dynamics simulations at 1 bar with isotropic position scaling and a pressure relaxation time of 2.0 ps throughout a MD simulation in 500 ps. Finally, an NPT simulation without any other constraint is implemented for 1 ns. All minimizations and dynamic simulations are performed with a dielectric constant of unity and a cut-off value for non-bonded pair interactions of 10.0 Å. Throughout the equilibration NVT simulations, the positions of the solute crystal model's heavy atoms are constrained with a relatively weak force of 10 kcal·mol^−1^. Throughout the equilibration NVT simulations, the positions of the solute crystal model's heavy atoms are constrained with a relatively weak force of 10 kcal·mol^−1^. The SHAKE option^40^ is adopted for bond interactions involving hydrogen atoms during dynamics calculations. All of the MD simulations are performed using the PMEMD module of the AMBER 12 package^41^ combined with the Glycam06^42^ carbohydrate parameter set. The MD trajectories are analyzed by the PTRAJ module of the AMBER 12 package and visualized using Visual Molecular Dynamics (VMD) 1.9.1 software^43^.

In the weak interaction theory, a variety of methods can be used to analyze the HBs interaction^39,44^. Recently, a new method has shown that the bonding critical point (BCP) has a linear relationship with HB strength^45^. It defines the calculation of the HB energy (kcal·mol^−1^) as follows,

5 $${\text{y}} = - 223.08 \times \rho BCP(a.u.) + 0.7423$$

where *ρ* is the electron charge density at the BCPs of HB. Based on Bader's theory, topological analyses of the electron charge density *ρ* at the BCPs is performed using atoms-in molecules (AIM) analysis^46^ in Multiwfn program^47,48^, and then it is visualized by the VMD program. To obtain accurate HBs geometry, the ONIOM model^49^ is used to further optimize HB’s geometry after the MD simulation. The wB97XD functional, together with the def2-SVP^50^ basis set, is used as a high layer to optimize the involving HBs parts (two anhydroglucose involving HB are selected), and the UFF force field^51^ is used as a lower layer to describe the remaining parts. Then the optimized HB structure by ONIOM model is further studied by the AIM analysis to obtain the electron charge density *ρ* for the calculation of HB strength.

The isolated chain sheet models are initially optimized using the AMBER/Glycam06 parameters, followed by the DFT optimization with 3 × cello-tetramer chain sheet models for 6A12, 6B12 and 6O12. The reason that three cello-tetramer is considered is that the three-chain sheet models can well depict the interaction with the adjacent chain, the double of the three chains can form the crystal plane, which is reasonable to adopt a 3 × cello-tetramer chain in DFT calculation. Only the hydrogen atoms’ position with the added terminal methyl groups are optimized in Gaussian09^52^, while all other atoms are fixed in position. In the DFT optimization, the present study used B3LYP-D3BJ function is coupled with the 6-31g(d) basis set. The single-point energy of the optimized model is then calculated using the 6-31+g(d,p) basis set. Besides, the solvent effects are mimicked by using the Solvation Model Based on Density (SMD)^53^ in water. The binding energy ($\Delta E_{bind}$) between adjacent oligomers is calculated as the difference between the DFT energies of the total model $E_{total}$ and its separate parts ($E_{{1}}$ and $E_{{2}}$), as defined by the following equations.

6 $$\Delta E_{bind} = E_{total} - E_{i}$$

7 $$\Delta E_{sheet} = E_{i} + \Delta E_{bind}$$

where *i* = 1, 2.$E_{{1}}$ and $E_{{2}}$ represent the energy of the two different oligomers, respectively. The three-chain sheet models $E_{{2}}$ represent the interior oligomer’s energy and the remainder of the sheet, respectively. The stabilization energy of the chain sheet ($\Delta E_{sheet}$) is defined as the sum of $E_{{\text{i}}}$ and $\Delta E_{bind}$, which implies the total energy of the oligomer *i* with the contribution of neighbor chain interaction. The basis set superposition error (BSSE) is estimated using the counterpoise method^52,54^.

GFN-xTB is one of the most newly developed semi-empirical DFT methods developed by the Grimme team^55–57^. It is an e**X**tended **T**ight-**B**inding semi-empirical program package (xtb) specially designed to calculate reasonable **g**eometry, vibration **f**requency, and **n**oncovalent interaction (GFN). Here we adopt it for the fast computation of 6A6, 6A12, 6A19, and 10A12, respectively. The geometry minimized by Amber is used to further optimization by GFN2-xTB with the C and O atoms fixed underwater circumstances. All these simulations were implemented on the Tianhe-1A supercomputer^58,59^.

## Supplementary Information

Supplementary Information.
