# Author Correction: Theoretical study of cellulose II nanocrystals with different exposed facets

**DOI:** 10.1038/s41598-023-30478-2

**Published:** 2023-03-02

**Authors:** Can Leng, Kenli Li, Zean Tian, Yubing Si, Huang Huang, Junfeng Li, Jie Liu, Wei‑Qing Huang, Keqin Li

**Affiliations:** 1 Science and Technology On Parallel and Distributed Processing Laboratory, National University of Defense Technology, Changsha, 410073 China; 2 Laboratory of Software Engineering for Complex Systems, National University of Defense Technology, Changsha, 410073 China; 3 National Supercomputer Centerin , Changsha, Changsha, 410082 China; 4 College of Computer Science and Electronic Engineering, Hunan University, Changsha, 410082 China; 5 College of Chemistry, Zhengzhou University, Zhengzhou, 450001 China; 6 College of Chemistry and Chemical Engineering, and Henan Key Laboratory of Function‑Oriented Porous Materials, Luoyang Normal University, Luoyang, 471934 China; 7 Department of Applied Physics, School of Physics and Electronics, Hunan University, Changsha, 410082 China; 8 Department of Computer Science, State University of New York, New Paltz, NY 12561 USA

Correction to: *Scientific Reports* 10.1038/s41598-021-01438-5, published online 08 November 2021

The Acknowledgements section in the original version of this Article was incomplete.

“This work is partially supported by the National Key Research and Development Program of China (Grant Nos. 2017YFB0202104, 2018YFB0204301).”

now reads:

“This work is partially supported by the National Key Research and Development Program of China (Grant Nos. 2017YFB0202104, 2018YFB0204301) and is the key scientific research projects of University in Henan Province (Grant No. 20A150027)”

The original Article has been corrected.

