# Supplementary material for: Theoretical study of cellulose II nanocrystals with different exposed facets
Source: Sci Rep. 2021 Nov 8;11:21871. doi: 10.1038/s41598-021-01438-5 (PMC8576008; doi:10.1038/s41598-021-01438-5)
Supplement: Supplementary file 1 — Supplementary Information. [file 41598_2021_1438_MOESM1_ESM.pdf]

# Theoretical Study of Cellulose II Nanocrystals with Different Exposed Facets

**Can Leng[1,2,3], Kenli Li[3,4], Zean Tian[4*], Yubing Si[5**], Huang Huang[3], Junfeng Li[6], Jie Liu[1,2], Wei-Qing Huang[7], and Keqin Li[4,8***]**

[1]Science and Technology on Parallel and Distributed Processing Laboratory, National University of Defense Technology, Changsha 410073, China
[2]Laboratory of Software Engineering for Complex Systems, National University of Defense Technology, Changsha 410073, China
[3]National Supercomputer Center in Changsha, 410082, China
[4]College of Computer Science and Electronic Engineering, Hunan University, Changsha 410082, China
[5]College of Chemistry, Zhengzhou University, Zhengzhou, 450001, China
[6]College of Chemistry and Chemical Engineering, and Henan Key Laboratory of Function-Oriented Porous Materials, Luoyang Normal University, 471934 Luoyang, China
[7]Department of Applied Physics, School of Physics and Electronics, Hunan University, Changsha 410082, China
[8]Department of Computer Science, State University of New York, New Paltz, New York 12561, USA

*tianzean@hnu.edu.cn

**ybsi@zzu.edu.cn

***lik@newpaltz.edu

## Supplementary Materials:

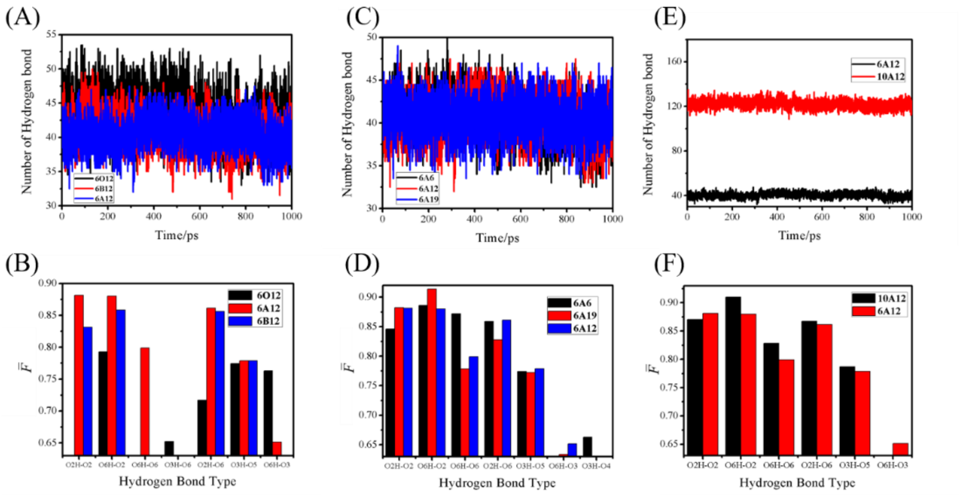


**Supplementary Figure S1. The number and average fraction $\overline{F}$ of hydrogen bonds in the cellulose II nanocrystal models at the terminal anhydroglucose.** (A) and (B) are the number and $\overline{F}$ of hydrogen bonds in 6O12, 6A12 and 6B12 of the terminal anhydroglucose, respectively; (C) and (D) are the number and $\overline{F}$ of hydrogen bonds in 6A6, 6A12 and 6A19 models of the terminal anhydroglucose, respectively; (E) and (F) are the number and $\overline{F}$ of hydrogen bonds in 6A12 and 10A12 of the terminal anhydroglucose, respectively.

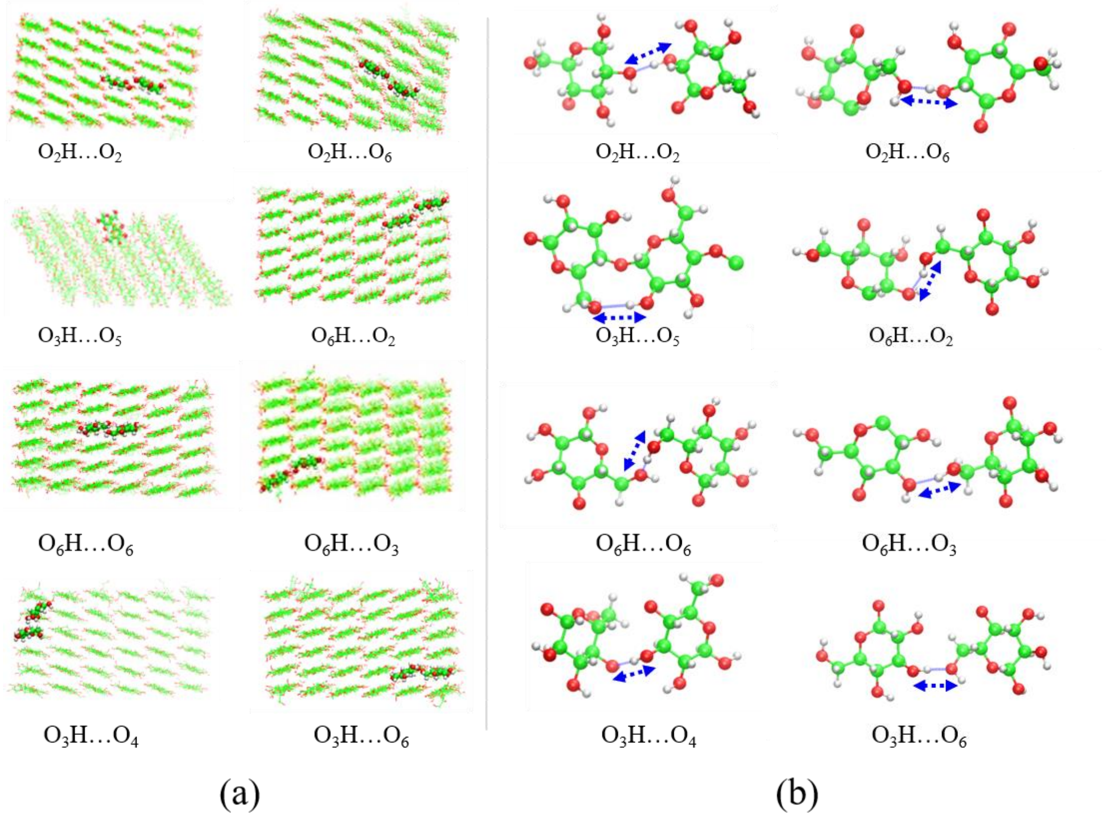


**Supplementary Figure S2. The ONIOM models and the hydrogen bonds scheme.** (A) The ONIOM models in which all hydrogen bond types are in their highest fraction samples; (B) The hydrogen bonds are colored with the blue dash line in cellulose II models.

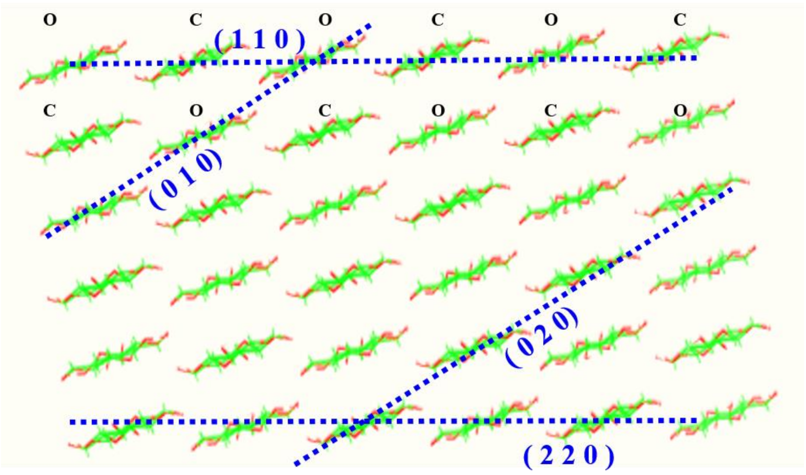


**Supplementary Figure S3. The 3×cello-tetramer chain sheet models derived from the original nanocrystal models of cellulose II.** O refers to the origin chain, C refers to the center chain. The (0 1 0), (0 2 0) and (1 1 0), (2 2 0) crystal plane are selected with a 3×cello-tetramer chain sheet in both O-C-O, C-O-C types.

**Supplementary Table S1. The average hydrogen bond analysis in six types of cellulose II nanocrystal models**. The type, average fraction $\bar{F}$ of HBs at the terminal residues, the average O···O distance (AvgDist) and angle (AvgAng) of HB type of the six nanocrystal models.

| Models | Type | $\bar{F}$ | AvgDist/Å | AvgAng/° |
|---|---|---|---|---|
| 6A6 | $O_2H\cdots O_2$ | 0.846 | 2.766 | 162.257 |
| | $O_6H\cdots O_2$ | 0.886 | 2.756 | 160.932 |
| | $O_6H\cdots O_6$ | 0.872 | 2.777 | 162.457 |
| | $O_2H\cdots O_6$ | 0.859 | 2.769 | 160.015 |
| | $O_3H\cdots O_5$ | 0.774 | 2.756 | 156.305 |
| | $O_3H\cdots O_4$ | 0.663 | 2.771 | 160.359 |
| 6A12 | $O_2H\cdots O_2$ | 0.881 | 2.764 | 162.561 |
| | $O_6H\cdots O_2$ | 0.880 | 2.755 | 160.982 |
| | $O_6H\cdots O_6$ | 0.799 | 2.782 | 162.645 |
| | $O_2H\cdots O_6$ | 0.861 | 2.769 | 159.775 |
| | $O_3H\cdots O_5$ | 0.779 | 2.752 | 155.918 |
| | $O_6H\cdots O_3$ | 0.652 | 2.761 | 161.520 |
| 6A19 | $O_2H\cdots O_2$ | 0.882 | 2.763 | 162.709 |
| | $O_6H\cdots O_2$ | 0.914 | 2.751 | 161.521 |
| | $O_6H\cdots O_6$ | 0.779 | 2.777 | 162.669 |
| | $O_2H\cdots O_6$ | 0.828 | 2.767 | 159.719 |
| | $O_3H\cdots O_5$ | 0.772 | 2.746 | 155.479 |
| | $O_6H\cdots O_3$ | 0.634 | 2.804 | 159.806 |
| 10A12 | $O_2H\cdots O_2$ | 0.871 | 2.764 | 162.392 |
| | $O_6H\cdots O_2$ | 0.910 | 2.754 | 161.209 |
| | $O_6H\cdots O_6$ | 0.828 | 2.776 | 162.800 |
| | $O_2H\cdots O_6$ | 0.867 | 2.765 | 159.811 |
| | $O_3H\cdots O_5$ | 0.787 | 2.750 | 155.662 |
| 6B12 | $O_2H\cdots O_2$ | 0.831 | 2.769 | 162.068 |
| | $O_6H\cdots O_2$ | 0.858 | 2.758 | 160.197 |
| | $O_2H\cdots O_6$ | 0.856 | 2.768 | 160.909 |
| | $O_3H\cdots O_5$ | 0.779 | 2.763 | 156.275 |
| 6O12 | $O_6H\cdots O_2$ | 0.793 | 2.757 | 161.494 |
| | $O_3H\cdots O_6$ | 0.652 | 2.798 | 158.006 |
| | $O_2H\cdots O_6$ | 0.717 | 2.791 | 157.030 |
| | $O_3H\cdots O_5$ | 0.774 | 2.768 | 155.736 |
| | $O_6H\cdots O_3$ | 0.763 | 2.740 | 159.833 |

**Supplementary Table S2.** Hydrogen bond type in different cellulose II nanocrystal models.

| | **6A6** | **6A12** | **6A19** | **10A12** | **6B12** | **6O12** |
|---|---|---|---|---|---|---|
| $O_2H\ldots O_2$ | ● | ● | ● | ● | ● | |
| $O_2H\ldots O_6$ | ● | ● | ● | ● | ● | ● |
| $O_3H\ldots O_5$ | ● | ● | ● | ● | ● | ● |
| $O_6H\ldots O_2$ | ● | ● | ● | ● | ● | ● |
| $O_6H\ldots O_6$ | ● | ● | ● | ● | | |
| $O_6H\ldots O_3$ | | ● | ● | | | ● |
| $O_3H\ldots O_4$ | ● | | | | | |
| $O_3H\ldots O_6$ | | | | | | ● |
